# StressGenePred: a twin prediction model architecture for classifying the stress types of samples and discovering stress-related genes in arabidopsis

**DOI:** 10.1186/s12864-019-6283-z

**Published:** 2019-12-20

**Authors:** Dongwon Kang, Hongryul Ahn, Sangseon Lee, Chai-Jin Lee, Jihye Hur, Woosuk Jung, Sun Kim

**Affiliations:** 1 Department of Computer Science and Engineering, Seoul National University, Seoul, Republic of Korea; 2 Interdisciplinary Program in Bioinformatics, Seoul National University, Seoul, Republic of Korea; 3 Department of Crop Science, Konkuk University, Seoul, Republic of Korea; 4 Bioinformatics Institute, Seoul National University, Seoul, Republic of Korea

**Keywords:** Arabidopsis, Stress, Transcriptome, Time-series, Machine learning

## Abstract

**Background:**

Recently, a number of studies have been conducted to investigate how plants respond to stress at the cellular molecular level by measuring gene expression profiles over time. As a result, a set of time-series gene expression data for the stress response are available in databases. With the data, an integrated analysis of multiple stresses is possible, which identifies stress-responsive genes with higher specificity because considering multiple stress can capture the effect of interference between stresses. To analyze such data, a machine learning model needs to be built.

**Results:**

In this study, we developed StressGenePred, a neural network-based machine learning method, to integrate time-series transcriptome data of multiple stress types. StressGenePred is designed to detect single stress-specific biomarker genes by using a simple feature embedding method, a twin neural network model, and Confident Multiple Choice Learning (CMCL) loss. The twin neural network model consists of a biomarker gene discovery and a stress type prediction model that share the same logical layer to reduce training complexity. The CMCL loss is used to make the twin model select biomarker genes that respond specifically to a single stress. In experiments using Arabidopsis gene expression data for four major environmental stresses, such as heat, cold, salt, and drought, StressGenePred classified the types of stress more accurately than the limma feature embedding method and the support vector machine and random forest classification methods. In addition, StressGenePred discovered known stress-related genes with higher specificity than the Fisher method.

**Conclusions:**

StressGenePred is a machine learning method for identifying stress-related genes and predicting stress types for an integrated analysis of multiple stress time-series transcriptome data. This method can be used to other phenotype-gene associated studies.

## Background

Recently, cellular molecule measurement technologies, such as microarray [1] and RNA-seq [2], can be used to measure the expression levels of tens of thousands of genes in a cell. Using these technologies, biologists have measured the change in gene expression levels under stress treatment over time. These time-series data are now available in databases such as ArrayExpress [3] and GEO [4]. To analyze of time-series transcriptome data, various methods were developed based on machine learning techniques such as linear regression, principal component analysis (PCA), naive Bayes, k-nearest neighbor analysis [5], simple neural network [6, 7], naive Bayes methods [8], and ensemble model [9].

However, existing methods were designed to analyze gene expression data of a single stress, not of multiple stresses. Analyzing gene expression data of multiple stresses can identify stress-responsive genes with higher specificity because it can consider the effect of interference between stresses. However, since no method of integrating multiple stress gene expression data has been developed, this study aims to develop a method for an integrated analysis of transcriptome of multiple stress types.

## Motivation

For the integrated analysis of transcriptome data of multiple stress, heterogeneous time-series analysis is should be considered [10]. Heterogeneous time-series analysis is a problem to analyze four-dimensional data of experimental condition (sample tissue, age, etc.), stress, time, and gene, where experimental condition axis and time axis are different among multiple time-series samples. Heterogeneous time-series analysis is explained in detail in the next section.

Many algorithms have been developed to analyze gene expression data. However, as far as we are aware of, there is no readily available machine learning algorithm for predicting stress types and detecting stress-related genes from multiple heterogeneous time-series data. Support vector machine (SVM) models are known to be powerful and accurate for classification tasks. Recently, SVMs are extended for multi-class problems and also for regression prediction. However, applying SVM for predicting stress-related genes and associating with phenotypes is not simple since the essence of the problem is to select small number of genes relevant to a few phenotypes. In fact, there is no known readily available prediction method for this research problem. Principal component analysis (PCA) is designed for predicting traits from the same structured input data, but it is not designed to analyze heterogeneous time-series data. Random forest (RF) is a sparse classification method, so how significant a gene is associated with stress is hard to be evaluated. Naive Bayes method [8] can measure the significance of genes, but it is not suitable for heterogeneous time-series data input. Clustering is one of the widely used machine learning approaches for gene expression data analysis. The STEM clustering method [11] clusters genes according to changes in expression patterns in time-series data analysis, but does not accept heterogeneous time-domain structure data.

Thus, we designed and implemented a neural network model, StressGenePred, to analyze heterogeneous time-series gene expression data of multiple stresses. Our model used feature embedding methods to address the heterogeneous structure of data. In addition, the analysis of heterogeneous time-series gene expression data, on the computational side, is associated with the *high-dimension and low-sample-size data problem*, which is one of the major challenges in machine learning. The data consists of a large number of genes (roughly 20,000) and a small number of samples (about less than 100). To deal with the high-dimension and low-sample-size data problem, our model is designed to share a core neural network model between twin sub-neural network models: 1) biomarker gene discovery model 2) stress type prediction model. These two submodels perform tasks known in the computer field as feature (i.e., gene) selection and label (i.e., stress type) classification, respectively.

## Materials

### Multiple heterogeneous time-series gene expression data

Multiple stress time-series gene expression data is a set of time-series gene expression data. The *k*-th time-series gene expression data, *D*_*k*_, contains expression values for three dimensional axes: gene axis, $G_{k}=\{g_{k1},\dots,g_{k|G_{k}|}\}$, time axis, $T_{k}=\{t_{k1},\dots,t_{k|T_{k}|}\}$, experimental condition axis, $F_{k}=\{f_{k1},\dots,f_{k|F_{k}|}\}$. However, the structure and values of time dimension and experimental condition dimension can be different in multiple samples, called “heterogeneous time-series data.”

1. **Heterogeneity of time dimension.** Each time-series data may have different number of time points and intervals.
2. **Heterogeneity of experimental condition dimension.** Each time-series data may have different experimental conditions, such as tissue, temperature, genotype, etc.

### The time-series gene expression datasets of four stress types

**Fig. 1:**
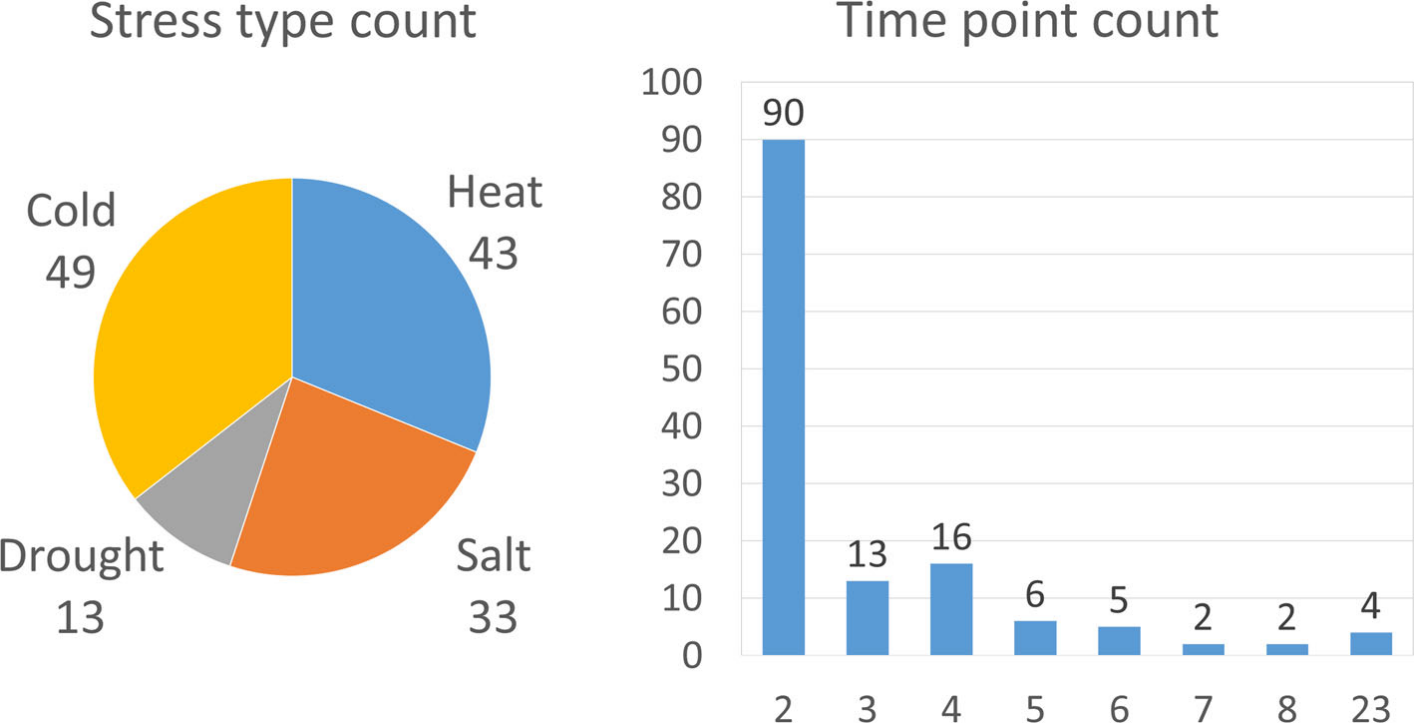
Dataset statistic summary. The number of stress types (left) and the frequency of time points (right) in the 138 sample time-series gene expression data of four stress types

In this paper, we analyze multiple heterogeneous time-series data of four major environmental stresses: heat, cold, salt and drought. We collected the 138 sample time-series data related to the four types of stress from ArrayExpress [3] and GEO [4]. Figure 1 shows the statistics of the collected dataset. The total dataset includes 49 cold, 43 heat, 33 salt, and 13 drought stress samples, and 65% of the time-series data are measured at only two time points. Every time point in each time-series data contains at least two replicated values.

## Methods

**Fig. 2:**
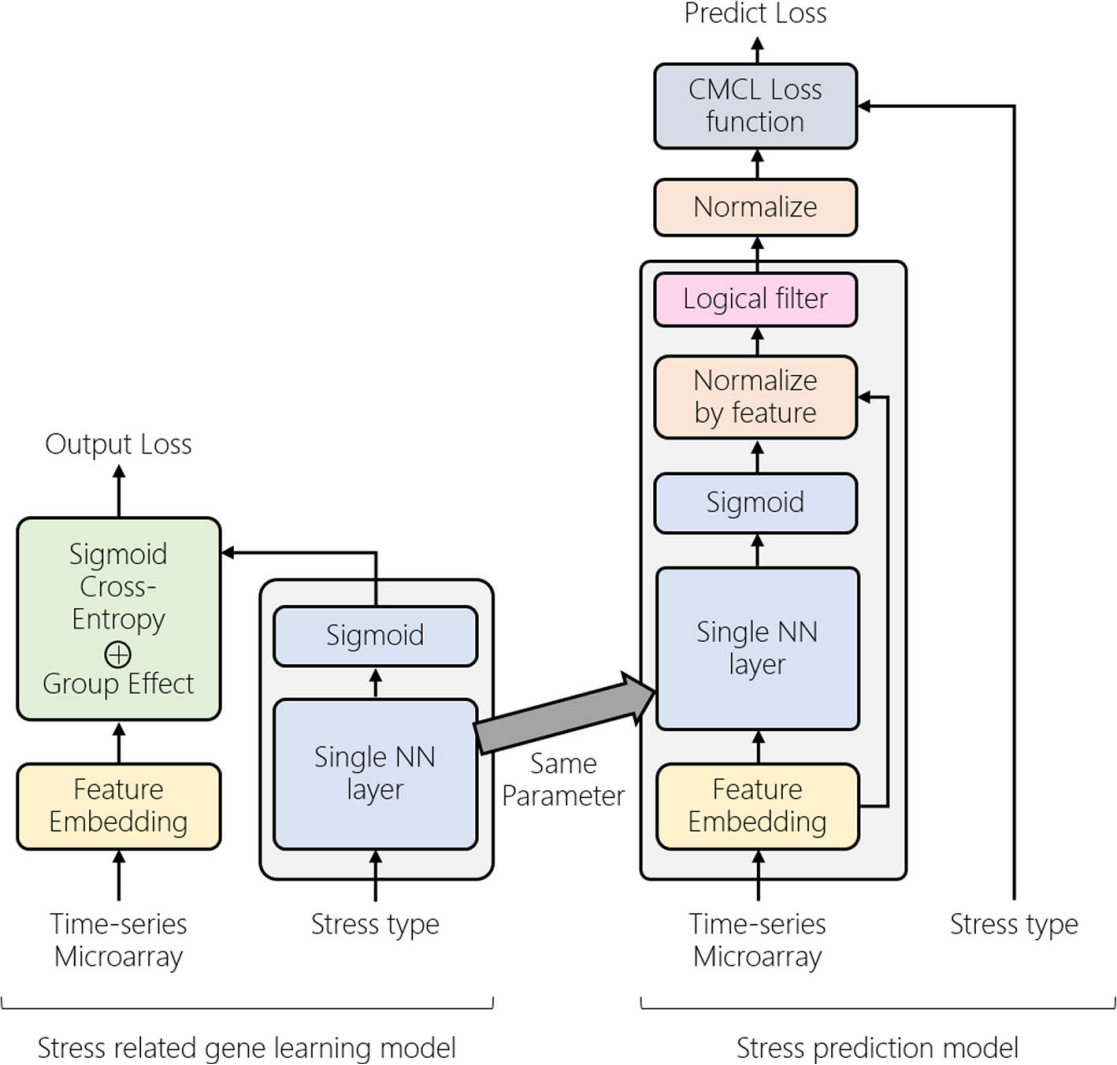
StressGenePred’s twin neural network model architecture. The StressGenePred model consists of two submodels: a biomarker gene discovery model (left) and a stress type prediction model (right). The two submodels share a “single NN layer”. Two gray boxes on the left and right models output the predicted results, biomarker gene and stress type, respectively

**Fig. 3:**
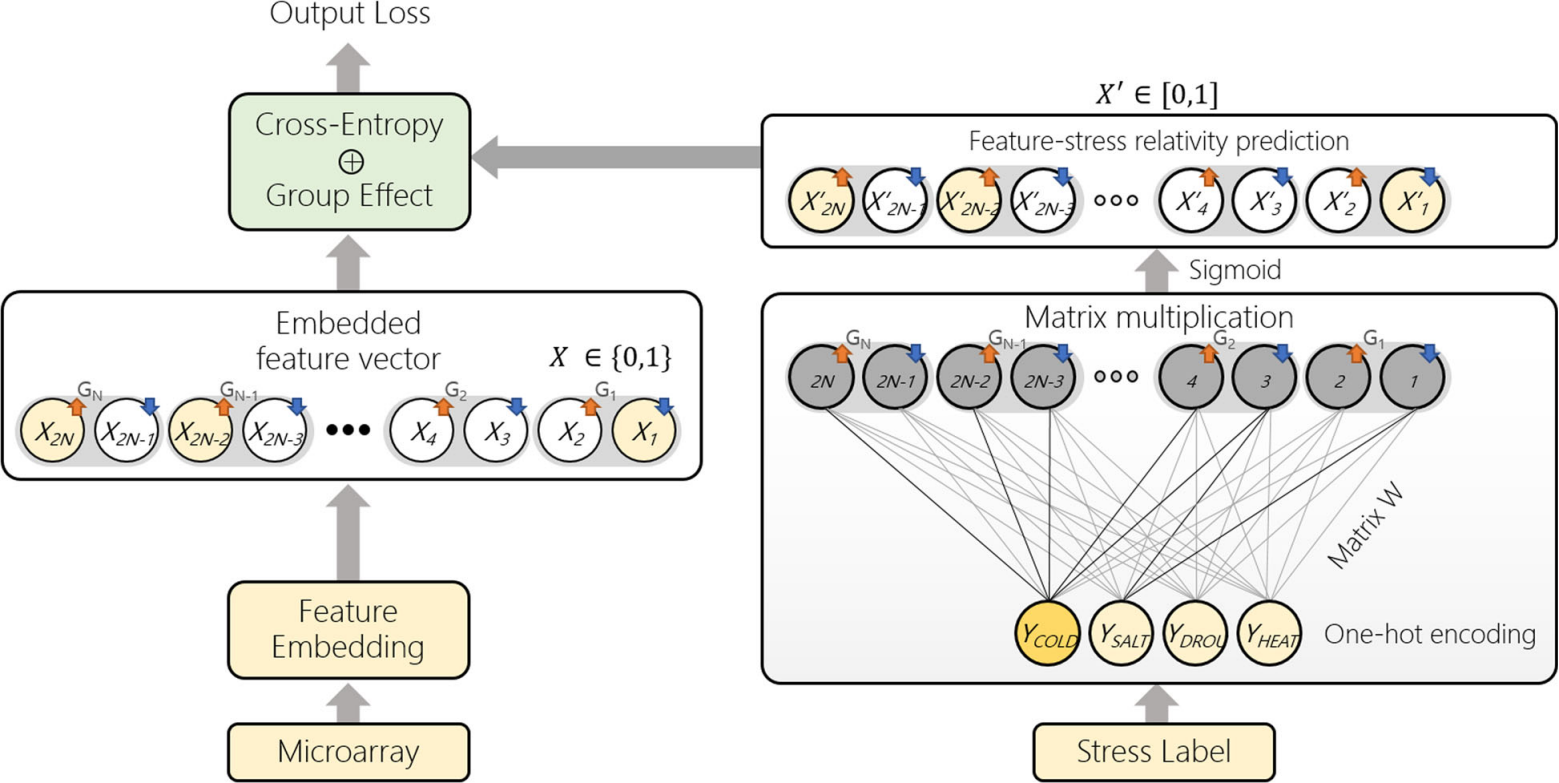
Biomarker gene discovery model. This model predicts biomarker genes from a label vector of stress type. It generates an observed biomarker gene vector from gene expression data (left side of the figure) and a predicted biomarker gene vector from stress type (right side of the figure), and adjusts the weights of the model by minimizing the difference (“output loss” at the top of the figure)

**Fig. 4:**
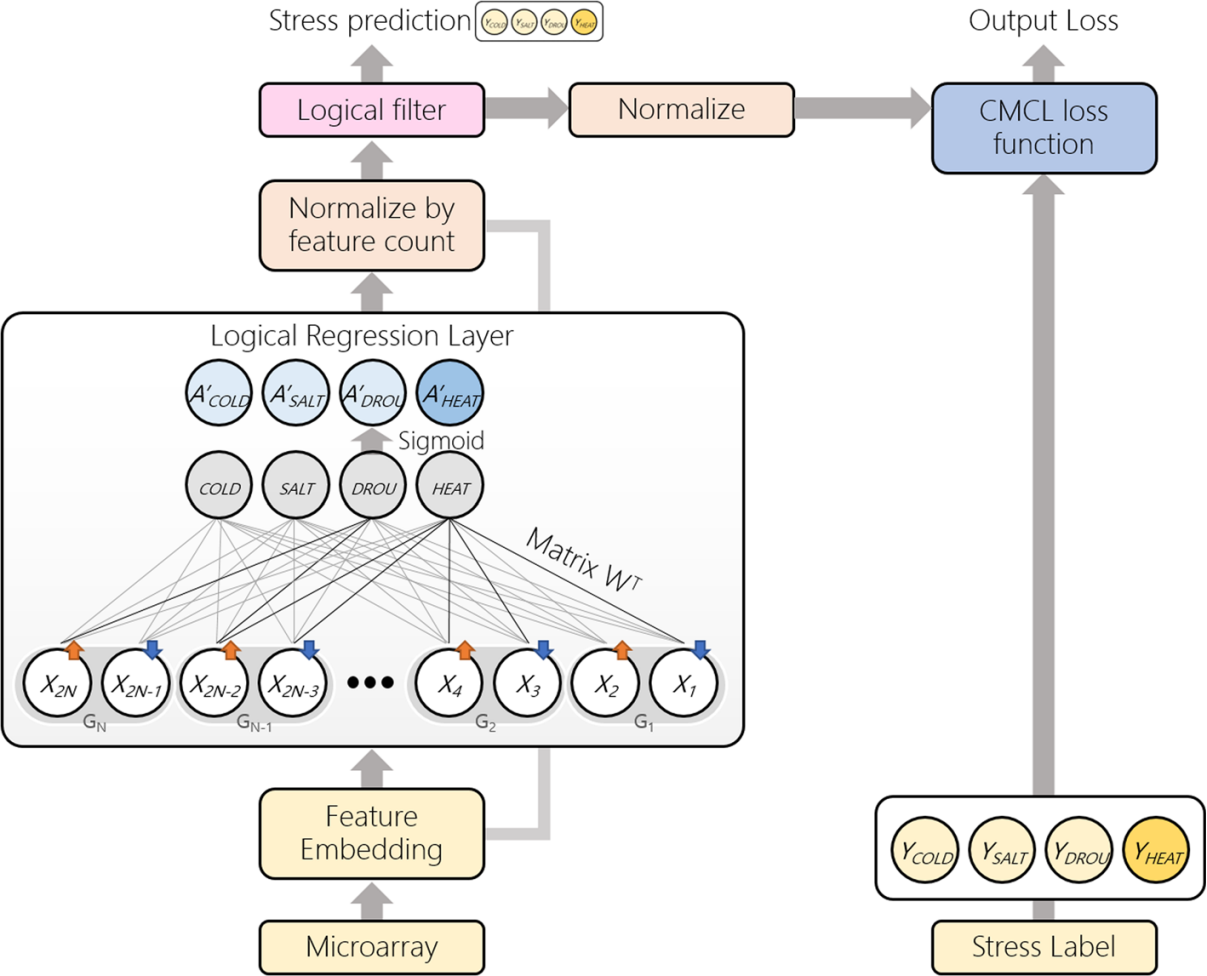
Stress type prediction model. This model predicts stress types from a vector of gene expression profile. It generates a predicted stress type vector (left side of the figure) and compares it with a stress label vector (right side of the figure) to adjust the weights of the model by minimizing the CMCL loss (“output loss” at the top of the figure)

StressGenePred is an integrated analysis method of multiple stress time-series data. StressGenePred (Fig. 2) includes two submodels : a biomarker gene discovery model (Fig. 3) and a stress type prediction model (Fig. 4). To deal with the high-dimension and low-sample-size data problem, both models share a logical correlation layer with the same structure and the same model parameters. From a set of transcriptome data measured under various stress conditions, StressGenePred trains the biomarker gene discovery model and the stress type prediction model sequentially.

### Submodel 1: biomarker gene discovery model

This model takes a set of stress labels, *Y*, and gene expression data, *D*, as input, and predicts which gene is a biomarker for each stress. This model consists of three parts: generation of an observed biomarker gene vector, generation of a predicted biomarker gene vector, and comparison of the predicted vector with the label vector. The architecture of the biomarker gene discovery model is illustrated in Fig. 3, and the process is described in detail as follows.

#### Generation of an observed biomarker gene vector

This part generates an observed biomarker vector, *X*_*k*_, from gene expression data of each sample *k*, *D*_*k*_. Since each time-series data is measured at different time points under different experimental conditions, a time-series gene expression data must be converted into a feature vector of the same structure and the same scale. This process is called feature embedding. For the feature embedding, we symbolize the change of expression before and after stress treatment by up, down, or non-regulation. In detail, a time-series data of sample *k* is converted into an observed biomarker gene vector of length 2*n*, *X*_*k*_={*x*_*k*1_,…,*x*_*k*2*n*_}, where *x*_*k*2*n*−1_∈{0,1} is 1 if gene *n* is down-regulation or 0 otherwise, *x*_*k*2*n*_∈{0,1} is 1 if gene *n* is up-regulation or 0 otherwise. For determining up, down, or non-regulation, we use the fold change information. First, if there are multiple expression values measured from replicate experiments at a time point, the mean of expression values is calculated for the time point. Then, the fold change value is computed by dividing the maximum or minimum expression values for a time-series data by the expression value at first time point. After that, the gene whose fold change value >0.8 or <1/0.8 is considered as up or down regulation gene. The threshold value of 0.8 is selected empirically. When the value of 0.8 is used, the fold change analysis generates at least 20 up or down regulation genes for all time-series data.

#### Generation of a predicted biomarker gene vector

This part generates a predicted biomarker gene vector, $X^{\prime }_{k}$, from stress type label *Y*_*k*_. $X^{\prime }_{k}=\{x^{\prime }_{k1}, \ldots, x^{\prime }_{2kn}\}$ is a vector of the same size as the observed biomarker gene vector *X*_*k*_. The values of *X*_*k*_*`* means up or down regulation as same as *X*_*k*_. For example, *x*_*k*2*n*−1_=1 means gene *n* is predicted as a down-regulated biomarker, or *x*_*k*2*n*_=1 means gene *n* is predicted as a up-regulated biomarker, for a specific stress *Y*_*k*_.

A logical stress-gene correlation layer, *W*, measures the weights of association between genes and stress types. The predicted biomarker gene vector, $X_{k}^{\prime }$, is generated by multiplying stress type of sample *k* and the logical stress-gene correlation layer, i.e., *Y*_*k*_×*W*. In addition, we use the sigmoid function to summarize the output values between 0 to 1. The stress vector, *Y*_*k*_, is encoded as one-hot vector of *l* stresses, where each element indicates whether the sample *k* is each specific stress type or not. Finally, the predicted biomarker gene vector, $X_{k}^{\prime }$, is generated like below:

$$\begin{array}{*{20}l} X^{\prime}_{k} = sigmoid(Y_{k} \times W) &= \frac{1}{1+exp(-Y_{k} \times W)} \\[0.4em] where ~~ W &= \left(\begin{array}{llll} w_{11} & w_{12} & \ldots & w_{1n} \\ \ldots & \ldots & \ldots & \ldots \\ w_{l1} & w_{l2} & \ldots & w_{ln} \end{array}\right) \end{array} $$

The logical stress-gene correlation layer has a single neural network structure. The weights of the logical stress-gene correlation layer are learned by minimizing the difference between observed biomarker gene vector, *X*_*k*_, and predicted biomarker gene vector, $X^{\prime }_{k}$.

#### Comparison of the predicted vector with the label vector

Cross-entropy is a widely-used objective function in logistic regression problem because of its robustness to outlier-including data [12]. Thus, we use cross-entropy as the objective function to measure the difference of observed biomarker gene vector, *X*_*k*_, and predicted biomarker gene vector, $X^{\prime }_{k}$, as below:

$$\begin{array}{*{20}l} loss_{W} = & - \sum\limits^{K}_{k=1} \left(X_{k} log (sigmoid(Y_{k}W)) \right.\\[-0.5em] &\left. \;\; + (1 - X_{k}) log (1-sigmoid(Y_{k}W)) \right) \end{array} $$

By minimizing the cross-entropy loss, logistic functions of the output prediction layer are learned to predict the true labels. Outputs of logistic functions can predict that a given gene responds to only one stress or to multiple stresses. Although it is natural for a gene to be involved in multiple stresses, we propose a new loss term because we aim to find a biomarker gene that is specific to a single stress. To control relationships between genes and stresses, we define a new group penalty loss. For each feature weight, the penalty is calculated based on how much stresses are involved. Given a gene *n*, a stress vector *g*_*n*_ is defined as *g*_*n*_=[*g*_*n*1_,*g*_*n*2_,...,*g*_*nl*_] with *l* stresses and *g*_*nl*_=*max*(*w*_*l*,2*n*_,*w*_*l*,2*n*+1_). Then, the a group penalty is defined as $(\sum (g_{n}))^{2}$. Since we generate the output with a logistic function, *g*_*nl*_ will have a value between 0 and 1. In other words, if *g*_*n*_ is specific to a single stress, the group penalty will be 1. However, if the gene *n* reacts to multiple stresses, the penalty value will increase quickly. Using these characteristics, the group penalty loss is defined as below:

$$loss_{group} = \alpha \sum\limits^{N}_{n=1} \left(\sum\limits^{L}_{l=1} g_{nl}\right)^{2}$$

On the group penalty loss, hyper-parameter *α* regulates effects of group penalty terms. Too large *α* imposes excessive group penalties, so genes that respond to multiple stresses are linked only to a single stress. On the other hand, if the *α* value is too small, most genes respond to multiple stresses. To balance this trade-off, we use well-known stress-related genes to allow our model to predict the genes within the top 500 biomarker genes at each stress. Therefore, in our experiment, the *α* was set to 0.06, and the genes are introduced in “Ranks of biomarker genes and the group effect for gene selection” section.

### Submodel 2: stress type prediction model

From biomarker gene discovery model, the relationships between stresses and genes are obtained by stress-gene correlation layer *W*. To build stress type prediction model from feature vectors, we utilize the transposed logical layer *W*^*T*^ and define a probability model as below:

$$A_{k} = sigmoid \left(X_{k} W^{T}\right)$$

$$A_{kl} = sigmoid \left(\sum\limits^{N}_{i=1} x_{ki} w_{il} \right) $$

Matrix *W* is calculated from a training process of the biomarker gene discovery model. *A*_*k*_ means an activation value vector of stress types, and it shows very large deviations depending on the samples. Therefore, normalization is required and performed as below:

$$A^{norm}_{k} = \frac{A_{k}}{\sum\limits^{N}_{n}{x_{kn}}} $$

For the logistic filter, these normalized embedded features vectors encapsulate average weight stress-feature relationship values that reduce variances among the vectors with different samples. As another effect of the normalization, absolute average weights are considered rather than relative indicator like softmax. So, false positive rates of predicted stress labels can be reduced. Using the normalized weights $A^{norm}_{k}$, logistic filter is defined to generate a probability as below:

$$g_{k}(A^{norm}_{k}) = \frac{1}{1+b_{l} \times exp(A^{norm}_{k}-a_{l})} $$

where *a* and *b* are general vector parameters of size *L* of logistic model *g*(*x*).

Learning of this logistic filer layer is started with normalization of the logistic filter outputs. This facilitates learning by regularizing the mean of the vectors. Then, to minimize loss of positive labels and entropy for negative labels, we adopted the Confident Multiple Choice Learning(CMCL) loss function [13] for our model as below:

$$\begin{array}{*{20}l} loss_{CMCL} &(Y_{k}, g(A^{norm}_{k})) = \\ &\sum\limits^{K}_{k=1} \left((1-A^{norm}_{k})^{2} - \beta \sum\limits^{L}_{l \neq Y_{k}} log(A^{norm}_{k}) \right) \end{array} $$

To avoid overfitting, a pseudo-parameter *β* is set by recommended setting from the original CMCL paper [13]. In our experiments, *β*=0.01≈1/108 is utilized.

## Results

In this paper, two types of experiments were conducted to evaluate the performance of StressGenePred.

### Evaluation of stress type prediction

StressGenePred was evaluated for the task of stress type prediction. The total time-series dataset (138 samples) was divided randomly 20 times to build a training dataset (108 samples) and a test dataset (30 samples). For the training and test datasets, a combination analysis was performed between two feature embedding methods (fold change and limma) and three classification methods (StressGenePred, SVM, and RF). The accuracy measurement of the stress type prediction was repeated 20 times.

Table 1 shows that feature embedding with fold change is more accurate in the stress type prediction than limma. Our prediction model, StressGenePred, more correctly predicted the stress types compared to other methods.

**Table 1:** Result of stress type prediction

Methods	Accuracy
StressGenePred+FC	0.963
RF+FC	0.961
SVM+FC	0.945
StressGenePred+limma	0.821
RF+limma	0.853
SVM+limma	0.813

Three stress type prediction models, StressGenePred (our model), random forest (RF) and support vector machine (SVM), are compared combined with two feature embedding models, fold change (FC) and limma

**Fig. 5:**
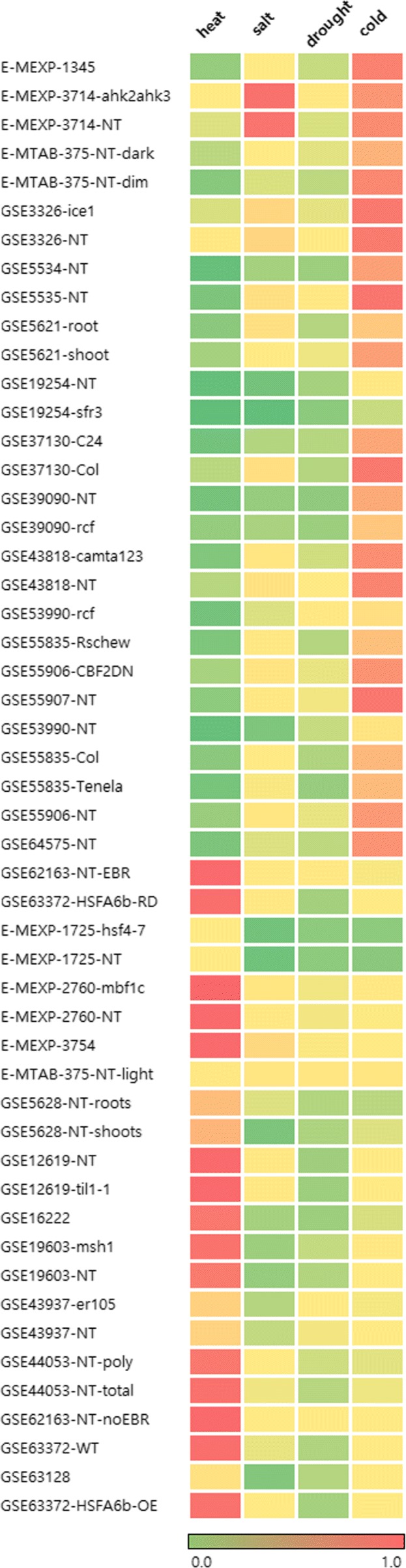
Stress type prediction result. Above GSE64575-NT are cold stress samples and the rest are heat stress samples. E-MEXP-3714-ahk2ahk3 and E-MEXP-3714-NT samples are predicted wrong in our model, but they are not perfectly predicted wrong because they are treated to both salt and cold stress [14]

Then, we further investigated in which cases our stress type prediction model predicted incorrectly. We divided the total dataset into 87 samples of training dataset and 51 samples of test dataset (28 cold stress and 23 heat stress samples). Then, we trained our model using training dataset and predicted stress types for the test dataset. Figure 5 shows three of 51 samples were predicted wrong in our model. Among them, two time-series data of cold stress type were predicted salt then cold stress types, and those samples were actually treated to both stresses [14]. This observation implied our prediction was not completely wrong.

### Evaluation of biomarker gene discovery

The second experiment was to test how accurately biomarker genes can be predicted. Our method was compared with Fisher’s method. The *p*-value of Fisher’s method was calculated using the limma tool for each gene for each stress types (heat, cold, drought, salt). The genes were then sorted according to their p-value scores so that the most responsive genes came first.

Then, we collected known stress-responsive genes of each stress type in a literature search, investigated EST profiles of the genes, and obtained 44 known biomarker genes with high EST profiles. We compared the ranking results of our method and Fisher method with the known biomarker genes. The Table 2 shows that 30 of 44 genes ranked higher in the results of our method than the Fisher method. Our method was better in the biomarker gene discovery than Fisher method (*p*=0.0019 for the Wilcoxon Signed-Rank test).

**Table 2:** Gene rank comparison

Stress type	Gene name	Gene symbol	Our method	Fisher method
Heat	**AT1G74310**	ATHSP101	2	11
	**ATMG00650**	NAD4L	5	44
	AT4G10250	ATHSP22.0	11	9
	AT4G27670	HSP21	12	7
	**AT1G16030**	Hsp70b	14	16
	**AT2G32120**	HSP70T-2	16	22
	AT4G25200	ATHSP23.6-MI	17	3
	**ATMG00070**	NAD9	19	54
	**AT5G09590**	MTHSC70-2	26	51
	AT5G12020	HSP17.6II	34	1
	AT5G37670		36	21
	AT2G26150	HSFA2	40	27
Cold	AT1G09350	AtGolS3	3	1
	**AT1G46768**	RAP2.1	4	29
	AT2G16890		18	16
	**AT5G17030**	UGT78D3	21	68
	**AT4G38580**	FP6	28	35
	**AT2G31360**	ADS2	35	116
	**AT1G23020**	FRO3	38	195
Salt	**AT3G02480**		1	2
	AT1G52690	LEA7	2	1
	AT5G59220	HAI7	4	4
	**AT5G06760**	AtLEA4-5	6	11
	**AT1G43160**	RAP2.6	10	12
	**AT4G05100**	AtMYB74	18	50
	**AT1G54100**	ALDH7B4	21	62
	**AT5G57050**	ABI2	23	77
	**AT5G13330**	Rap2.6L	26	36
	AT1G52890	NAC019	28	15
	**AT3G04070**	NAC047	29	73
	AT3G48520	CYP94B3	31	27
	**AT4G19230**	CYP707A1	33	75
	AT1G07430	HAI2	36	16
Drought	AT2G46680	ATHB-7	3	1
	**AT1G52890**	NAC019	4	15
	**AT3G03470**	CYP89A9	11	271
	**AT2G18050**	HIS1-3	12	21
	**AT1G29440**	SAUR63	13	53
	**AT4G22950**	AGL109	21	2002
	**AT4G32940**	GAMMA-VPE	23	426
	**AT1G18650**	PDCB3	25	778
	**AT1G56600**	GolS2	31	33
	**AT2G21650**	MEE3	38	855
	**AT4G30610**	BRS1	39	468

The 44 known biomarker genes with high EST profiles are collected. In comparison of our method (StressGenePred) with Fisher method, 30 of 44 known biomarker genes (bold) are ranked higher in the result of our method than the Fisher method

Our method is designed to exclude genes that respond to more than one stress whenever possible and to detect genes that only respond to one type of stress. To investigate how this works, we collected genes known to respond to more than one stress. Among them, we excluded genes that resulted in too low a ranking (>3,000) for all stress cases.

**Fig. 6:**
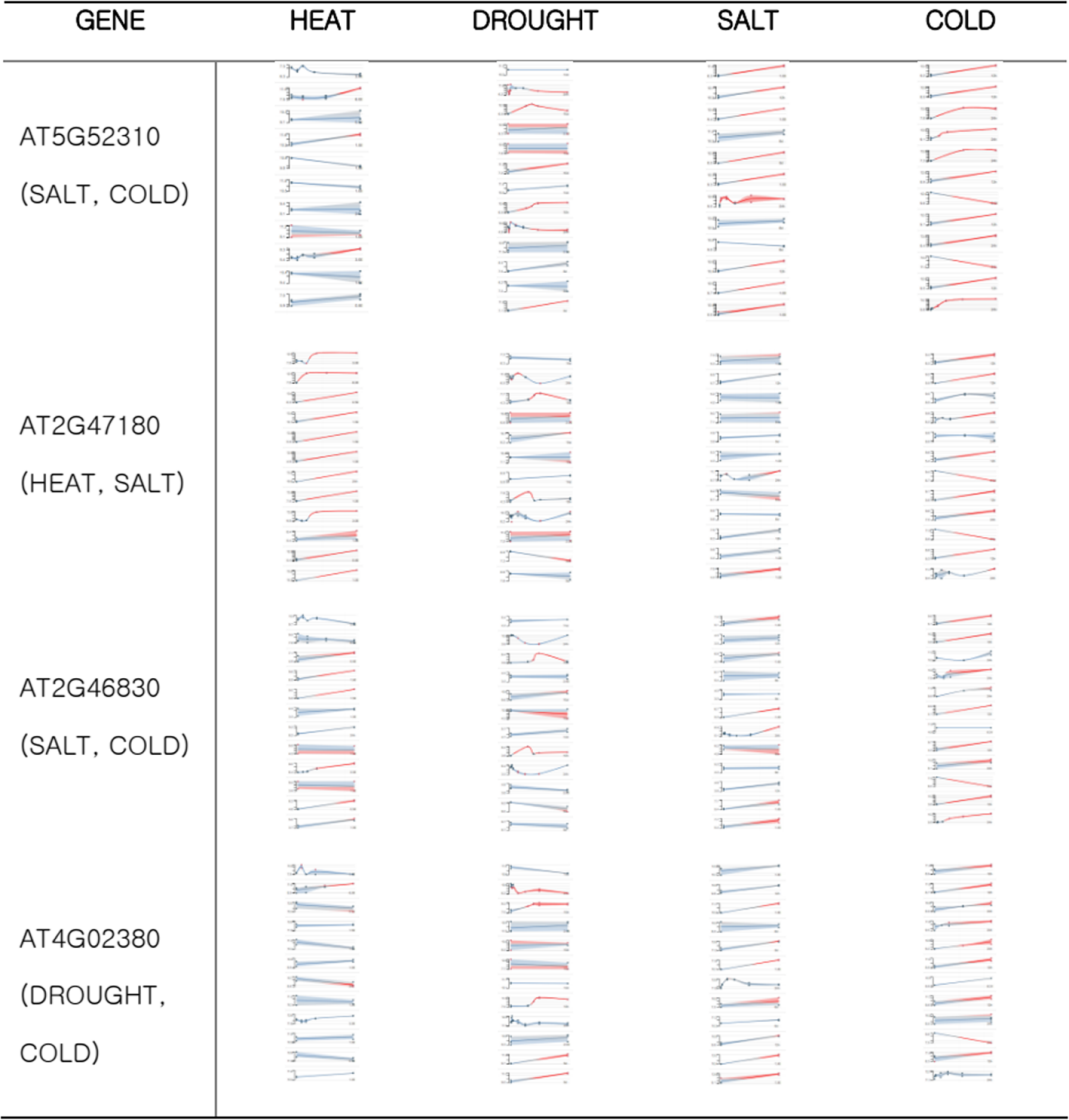
Visualization of gene expression for multiple stress associated genes. Genes that were investigated to be responsive to multiple stresses. In the visualization results, these genes responded to multiple stresses and were not suitable for biomarker genes of a single stress

When comparing the results of our method to the Fisher method for these genes, 13 of 21 genes ranked lower in the result of our method than Fisher method (Table 3). This suggests that our model detects genes that respond only to one type of stress. Figure 6 shows a plot of changes in expression levels of some genes for multiple stresses. These genes responded to multiple stresses in the figure.

**Table 3:** Rank comparison of multiple stress-responsive genes

Genename	GOTerm	Rank of our model	Rank of fisher method
AT2G47180	heat,cold	heat(243), cold(500)	heat(39), cold(164)
AT5G37770	heat,cold	heat(2007), cold(3414)	heat(1878), cold(2510)
AT5G57560	heat,cold	heat(1357), cold(1428)	heat(235), cold(627)
AT5G58070	heat,cold	heat(693), cold(111)	heat(258), cold(167)
AT5G59820	heat,cold	heat(1069), cold(512)	heat(234), cold(128)
AT2G47180	heat,salt	heat(243), salt(842)	heat(39), salt(722)
AT3G09350	heat,salt	heat(61), salt(1341)	heat(35), salt(1712)
AT1G01060	cold,salt	salt(1762), cold(1342)	salt(1578), cold(298)
AT2G17840	cold,salt	salt(120), cold(247)	salt(279), cold(34)
AT2G19450	cold,salt	salt(1201), cold(86)	salt(700), cold(162)
AT2G38470	cold,salt	salt(234), cold(4958)	salt(142), cold(3504)
AT2G42540	cold,salt	salt(257), cold(79)	salt(538), cold(23)
AT2G46830	cold,salt	salt(506), cold(267)	salt(338), cold(31)
AT2G47180	cold,salt	salt(842), cold(500)	salt(722), cold(1642)
AT3G23830	cold,salt	salt(2516), cold(3530)	salt(1590), cold(2493)
AT3G48360	cold,salt	salt(1007), cold(1968)	salt(111), cold(447)
AT5G23860	cold,salt	salt(1280), cold(320)	salt(2527), cold(449)
AT5G52300	cold,salt	salt(43), cold(2982)	salt(38), cold(1327)
AT5G52310	cold,salt	salt(10), cold(333)	salt(6), cold(4)
AT5G58670	cold,salt	salt(291), cold(2148)	salt(634), cold(1284)
AT4G02380	cold,drought	drought(1013), cold(416)	drought(136), cold(278)

To investigate that StressGenePred excludes genes that respond to more than one stress, 21 genes known to respond to more than one stress are collected. Among the 21 genes, 13 genes rank lower in the result of StressGenePred than Fisher method (Table 3)

### Literature-based investigation for discovered biomarker genes

In order to evaluate whether our method found the biomarker gene correctly, we examined in literature the relevance of each stress type to the top 40 genes. Our findings are summarized in this section and discussed further in the discussion section.

In the case of heat stress, we identified heat-related genes, including HSFA2, which are known to play an essential role in the plant’s heat response. Heat shock protein genes such as HSP101, HSP15.7, HSP17.6, HSP20-like, Hsp21, Hsp22, Hsp70B, and Hsp70T-2 we have identified are known to be highly related to heat stress. Mitochondrial heat shock protein genes such as AtHSP23.6 and MTHSC70-2 and chloroplast position genes such as HSP21 have also been identified. We predicted NADH dehydrogenases of energy metabolism which are related to heat stress.

In the case of salt stress, we have identified previously known ABA-related genes, such as ABI2, ABF1, HAI1 and HAI2, and late embryonic development-rich protein genes, such as AtLEA4-5, LEA7. Water biomarker genes as ATD18, NAC019, NAC047 and RAP2.6 were identified. We have also identified genes of common stress-response class genes, such as ALDH7B4 and ALDH2B7, AtMYB74, CYP707A1, and CYP94B3.

In the case of cold stress, we identified ADS2, AtGolS3, FP6, FRO3, GSTU18, UDP-glucosyl transferase, some lipid metabolism-related genes that are involved in a rearrangement of physical properties of the plasma membrane and cell wall. In addition, we identified genes related to development such as AGL20, BBX29, and GI. We also identified water biomarker genes such as ABF1, BBX25, and RAP2.1.

Finally, in the case of drought stress, we confirmed the involvement of well-known genes such as HIS1-3, NAC019 and SAUR63. Besides, we were able to identify common biomarker genes such as development-related AGL19 and CYP89A9. In addition, we predicted genes involved in microorganism development and differentiation such as ATHB-7, BRS1, GAMMA-VPE, GOLS2, MEE3, and PDCB3.

## Discussion

In this section, we discuss gene-stress relationship in depth, referring to the current literature.

### Biological function of heat stress-responsive genes

For heat stress, our model identified HSFA2, Hsp21, Hsp22, Hsp70B, Hsp70T-2, HSP101, HSP20-like, HSP17.6, HSP15.7, and NADH dehydrogenases. In heat stress, HSFA2 takes an essential part of heat response and may relate with histone methylation. HSFA2 is highly inducible and a direct target of HSFA1. HSFA2 is known to bind to the promoter of Hsp22 in vitro experiments [15]. Hsp22 is an endomembrane-localized protein during heat stress [16]. Hsp70 family proteins are well-known proteins, however functionally diversified. Hsp21 is small heat shock protein, which required for the development of chloroplasts [17] and associates with the thylakoid membranes [18]. HSP70 is a molecular chaperone and support plastid protein translocation [19]. HSP70b may involve a protein accumulation in the cytosol [20] and inducible by heat shock, not by low temperature [21]. HSP101 is a member of the Hsp100/ClpB family of proteins, is thought to be involved in disaggregation of misfolded proteins [22]. HSP101 protects protein translation factors during heat stress [23]. HSP17.6 is induced by heat and osmotic stress, and overexpression of AtHSP17.6A increase salt and drought tolerance in Arabidopsis [24]. Hsp17.6CII is a peroxisome-localized catalase chaperone [23]. Also, HSP15.7 is inducible by heat shock and high light, detected in peroxisome [25]. Interestingly, both the chloroplast-located genes HSP21 and mitochondrial heat shock proteins such as AtHSP23.6 and MTHSC70-2 were identified.

### Biological function of cold stress-responsive genes

For cold stress, our model predicted many genes involved in plasma membrane fluidity and cell wall rigidity. ADS2 gene adjusts the composition of membrane lipids, and confer chilling and freezing tolerance in Arabidopsis [26]. AtGolS3 codes galactinol synthase 3 which is only induced by cold stress and target of DREB1A [27]. FP6 is farnesylated protein 6, interacts with ACBP2, and the transgenic plants showed overexpression had Cd(II) tolerance [28]. FRO is an iron chelate reductase, and FRO3 is predicted to involve in iron metabolism and iron reduction in the root [29].

### Biological function of salt stress-responsive genes

For salt stress, our model identified ABI2, ABF1, HAI1, HAI2, LEA7, AtLEA4-5, NAC019, NAC047, ATD18, RAP2.6, CYP707A1, CYP94B3, AtMYB74, ALDH7B4 and ALDH2B7 genes. In salt stress, many genes of downstream signal transduction or possibly related with ABA such as ABI2, ABF1, HAI1 and HAI2, late embryogenesis abundant proteins like LEA7 and AtLEA4-5. ABI2 is a protein phosphatase 2C, interacts with SOS2 and inhibits SOS2 activity [30]. ABI2 involved in ABA-mediated transcription of chloroplast genes and link nitrate uptake and utilization [31]. ABF1 regulates the induction of DREB2A [17] and is necessary for seedling establishment during winter. Expression of ABF1 is induced by cold, heat, and ABA [32]. HAI1 has roles in decreasing the low water potential signaling that controls proline and osmoregulatory solute accumulation [33]. HAI1 is involved in feedback regulation of ABA signaling and HAI2 is a positive regulator of ABA and related to cell signaling mediated by ABA [34]. Late embryogenesis abundant proteins like LEA7 could protect the plasma membrane or organellar membrane. Its activity occurs at cytosol exposed side of the membrane [35]. AtLEA4-5 is a member of small, hydrophilic protein group, showing high expression levels in response hyperosmotic, drought, and ABA treatment [36]. NAC is a water stress-responsive transcription factor. NAC019 has ABRE-like motifs, and the motifs could induce expression in response to stress. NAC019 promoter interacts with a key mediator of ABA expression, ABI4, AP2 family transcription factors [37]. ATD18, also known as RAB18, is dehydrin family protein and required for ABA signal transduction. ATD18 expression is repressed by ethylene treatment [38]. RAP2.6 is induced by salt and osmotic stress. RAP2.6 promoter contains ABRE, DRE, MYBR, W-box, RAVbox, so seems like it may be an essential intersection in biotic and abiotic signaling [39]. CYP707A1 is a member of cytochrome P450 CYP707A family encoding ABA-8’-hydroxylases. CYP707As are working as structure modifiers of metabolites responsive to the abiotic stress, exogenous ABA treatment, and dehydration [40].

### Biological function of drought stress-responsive genes

For drought stress, our model predicted many of early response genes against water stress. HIS1-3 has histone H1 globular domain and is expressed by dehydration and ABA [41]. SAUR63 is a member of early auxin-responsive genes family, promoting organ elongation by auxin stimulation in Arabidopsis [42]. AGL19 is expressed by a short-day photoperiod and vernalization [43]. Gamma-VPE is a type of vegetative VPE and induced during senescence, wounding, and pathogen infection [44]. Gamma-VPE has a cysteine protease activity and may be involved in plant hypersensitive cell death [41]. GOLS2 increase galactinol biosynthesis and improve oxidative stress tolerance. This gene regulated by HsfA3 [45]. AtGolS2-expressing transgenics displayed significantly improved drought tolerance [46]. MEE3 (Maternal Effect Embryo arrest 3) is a subfamily of single-MYB transcription factor and related to regulation of early photomorphogenesis [47]. BRS1 is involved in brassinosteroid signaling pathway. This gene was expressed strongly in the root and related to plant root development [48]. BRS1 gene encodes a serine carboxypeptidase II-like protein, secreted and active serine carboxypeptidase [49].

### Stress responsive transcription factors

We examined genes that change expression levels with respect to temperature stress. Some of these genes were transcription factors, and they did not appear for other type stress because our predictive model predicted genes specifically associated with specific stresses. But what we can observe is that TFs, such as ARF, ERF, bZIP, which are involved in plant hormonal reactions, can be activated at both high and low temperatures when there are temperature-related stresses. Our model predicted NAD4L and NAD5 (NADH dehydrogenase subunits 4L and 5) and several unknown genes encoded in the mitochondrial genome that only affected heat stress. Some genes in mitochondria may be involved in the initial transcriptional response when under heat stress. In the case of salt and drought stress, we predicted two TF genes, HD-ZIP (ATHB-5; AT2G468) and NAC (ANAC019: AT1G5289), which are associated with both stresses. These two genes are likely to respond early to water-related stress. NAC domain TF is prominent in salt stress, but not drought stress. We observed SAURs (small auxin upregulated RNA) in drought stress, which means that it is a small RNA that is actively involved in plant physiological regulation during long-term water deficiency.

### Diversity of responses to multiple stresses

In this study, we selected four different types of stress to find and classify the affected genes. The effects of these environmental stresses are overwhelming, but they do not define specific parts of metabolism and physiological consequences. The characteristics of the four stresses we studied have in common with the physiological response associated with water. Although they react differently depending on the signaling pathways of each stress, they do not have complete separation because of the commonalities associated with using water. Many of the biomarker genes we have found have been shown to respond to multiple stresses, and have shown a variety of phenotypes for different stresses in plants that have been transfected with mutations or recombinant genes. The APX gene is a gene that responds to all four stresses, and other genes such as AREB, AtRIP, DREB, Gols and MAPs are well known as genes that respond to multiple stresses. In this study, the genes involved in the specific stresses we predicted were either identical in other stresses or related to multiple complex stresses.

## Conclusion

This study presented StressGenePred, a method of analyzing a set of time-series transcriptome data for multiple types of stress. StressGenePred consists of twin classification models to achieve two analytic goals. The biomarker gene discovery model aims to discover genes that respond to specific stresses. The goal of the stress type prediction model is to classify samples into four types of stress, heat, cold, drought, and salt. The key problem in this study is to train the StressGenePred model from high-dimension (approximately 20,000 genes) and low-sample-size data (138 sample data in the study). Analysis of high-dimension and low-sample-size data is a difficult computational problem that many researchers are studying.

In order to be trained with a small number of data, StressGenePred is designed to use a simplified architecture (only one logical layer) with a small number of parameters. StressGenePred is also designed so that twin classification models share the same logical layer and its parameters. In twin classification models, the logical layer is used symmetrically with respect to input and output. For example, the input and output in the biomarker gene discovery model are stress and genes, respectively, and the stress type prediction model is vice versa. When the logical layer is shared by both classification models, the parameters of the logical layer are trained redundantly in both models, reducing the number of data required.

In experiments using Arabidopsis stressed gene expression data, StressGenePred detected known stress-related genes at a higher rank compared to Fisher’s method. StressGenePred showed better performance than random forest and support vector machine in stress type prediction.

## Data Availability

The data information and source codes of StressGenePred are available at https://github.com/bhi-kimlab/StressGenePred.

## Abbreviations

CMCL: Confident multiple choice learning
DEG: Differentially expressed gene
FC: Fold change
GEO: Gene expression omnibus
PCA: Principal component analysis
RF: Random forest
RNA-seq: Ribonucleic acid-sequencing
SVM: Support vector machine
